# A Review of Cases of Marijuana and Violence

**DOI:** 10.3390/ijerph17051578

**Published:** 2020-02-29

**Authors:** Norman S. Miller, Redon Ipeku, Thersilla Oberbarnscheidt

**Affiliations:** 1 CEO of Health Advocates PLLC, East Lansing, MI 48823, USA; 2 Department of Psychiatry Augusta University, Augusta, GA 30912, USA; 3 College of Law, Michigan State University, East Lansing, MI 48823, USA; redon.ipeku@gmail.com; 4 Department of Psychiatry, University of Pittsburgh Medical Center, Pittsburgh, PA 15213, USA; oberbarnscheidtt@upmc.edu

**Keywords:** marijuana, cannabis, tetrahydrocannabinol, THC, violence, law, paranoia, delusions, psychosis, public policies

## Abstract

Marijuana is the most consumed illicit drug in the world, with over 192 million users. Due to the current legalization push of marijuana in the United States, there has been a lack of oversight regarding its public health policies, as marijuana advocates downplay the drug’s negative effects. This paper’s approach is from a public health perspective, focusing specifically on the cases of violence amongst some marijuana users. Here, we present 14 cases of violence with chronic marijuana users that highlight reoccurring consequences of: marijuana induced paranoia (exaggerated, unfounded distrust) and marijuana induced psychosis (radical personality change, loss of contact with reality). When individuals suffering from pre-existing medical conditions use marijuana in an attempt to alleviate their symptoms, ultimately this worsens their conditions over time. Although marijuana effects depend on the individual’s endocannabinoid receptors (which control behavioral functions, like aggression) and the potency level of tetrahydrocannabinol (THC) in the drug, scientifically documented links between certain marijuana users and violence do exist. Wider public awareness of the risks and side effects of marijuana, as well as a more prudent health policy, and government agency monitoring of the drug’s composition, creation, and distribution, are needed and recommended.

## 1. Introduction

In the United States, ten states have legalized the recreational use of marijuana and over 20 states have decriminalized the recreational use of it. Recent reports suggest, however, that the increase of the recreational use of marijuana is causing detrimental effects to individuals, as well as the society as a whole [1,2,3]. These effects include, but are not limited to, the increase of violence, the increase of thriving underground marijuana markets, and increase in car accident claims after the legalization of marijuana where the recreational use of marijuana was legalized [1,2,3]. This is caused by lack of oversight. Marijuana is being legally sold with high THC concentration levels without taking into account its addictive qualities and adverse effects. On the other hand, and contrary to popular belief, marijuana is still illegal in the Netherlands and it is decriminalized. However, the consumption and storage of marijuana are limited by law and the approach taken by the Netherlands is to decriminalize the drug in order to be able to help individuals struggling with marijuana use. This prudent oversight has resulted in a decreased in violence and people are able to get the care they need to deal with addiction and become less prone to violence [1,2,3].

Furthermore, the consumption of marijuana is associated with an increase in violent behavior over the course of an individual’s lifespan, a high risk of psychosis for frequent users, an increase of cardiovascular diseases, and deterioration in health for individuals who have pre-existing mental health issues such as Post Traumatic Stress Disorder, social anxiety, and depression [4,5,6].

According to research studies, marijuana use causes aggressive behavior, causes or exacerbates psychosis, and produces paranoia. These effects have been illustrated through case studies of highly publicized incidents and heightened political profiles.

These cases contain examples of repeated illustrations of aggression, psychosis and paranoia by marijuana users and intoxication. Ultimately, without the use and intoxication of marijuana, the poor judgment and misperceptions displayed by these individuals would not have been present, reducing the risk for actions that result in senseless deaths.

Import to these assertions, is that the current marijuana is far more potent in THC concentrations, the psychoactive component. Accordingly, and demonstrated in direct studies, more potent marijuana results in a greater risk for paranoid thinking and psychosis. In turn, paranoid behavior increases the risk for paranoid behaviors and predictably associated with aggressive and violent behaviors.

1. Marijuana use causes violent behavior through increased aggressiveness, paranoia, and personality changes (more suspicious, aggressive, and anger).
2. Recent illicit and “medical marijuana” (especially grown by care givers for medical marijuana) is of much high potency and more likely to cause violent behavior.
3. Marijuana use and its adverse effects should be considered in cases of acts of violence as its role is properly assigned to its high association.
4. Recognize that high potency marijuana is a predictable and preventable cause of tragic violent consequences.

## 2. Case Presentation

### 2.1. Marijuana Violence

On March 13, 2019, Anthony Comello admitted to, and subsequently was charged for, the killing of Frank Cali, a senior leader of the Gambino family in Staten Island, New York. Both men were allegedly having an altercation over Comello’s romantic interest on one of Cali’s relatives. Although Comello had no previous criminal encounters with law enforcement, reports suggest that he drew the attention of authorities by acting strangely in a federal courthouse when he offered to perform a citizen’s arrest on New York City’s Mayor, Bill De Blasio. Previously, Comello sought a U.S. Marshal to inquire how to perform a citizen’s arrest on the United States Speaker of the House, Nancy Pelosi. Comello admitted that, at the time of Cali’s killing, he was high on marijuana and shot Cali because he feared that the senior leader had a gun and would shoot him during their altercation [4,7].

On February 10, 2019, a man killed his 13-year-old nephew with a knife in Rustavi, Georgia. The man had a history of marijuana use. For days leading up to the killing, he was complaining about having dizziness, headaches, general weakness, nausea, and insomnia. He would also occasionally suffer from anxiety, irritability, and loss of appetite. His wife stated that he consumed and was under the influence of marijuana, which made his symptoms worse. The day before the killing, he tried to go to a clinic. However, the clinic rejected treatment by telling him to go to a psychiatric hospital. However, under the influence of drugs, he simply went home hours before killing his nephew [8].

On February 1, 2018, Nikolas Cruz killed 17 students and staff at the Marjory Stoneman Douglas High School in Parkland, Florida, and injured 17 others. Cruz was diagnosed as developmentally delayed at age three and had numerous disciplinary issues dating to middle school. From a young age, he started consuming marijuana heavily. He accounts that he would frequently “hear demon voices” and would consume large amount of marijuana to try and silence those voices. He also attempted suicide. During an interview after his mass shooting, he stated that he used “a lot of marijuana” as well as prescription tranquilizer Xanax [9,10].

On November 5, 2017, Devin Patrick Kelley carried out the deadliest mass shooting in Texas’ history, resulting in the death of 27 people and injuries to 20 others, by opening fire at worshippers who were attending regular Sunday Service at the First Baptist Church in Sutherland Springs. Kelley was later shot by bystanders and killed during a high-speed chase with law enforcement agencies. The autopsy on Kelley revealed that toxicology tests detected marijuana and anti-anxiety drugs in his system. A report from the Federal Bureau of Investigation revealed multiple past incidents where Kelley also been under the influence of marijuana. Kelley’s first on-record interaction with law enforcement authorities was when he was arrested for possession of marijuana and, subsequently, expelled from his high school. Since then, the record shows that Kelley started using marijuana frequently, as well as developing mental health issues that would lead him to have problems in his employment with the United States Air Force and multiple instances where he abused his step-son and his wife at the time [11,12].

On May 22, 2017, a suicide bomber, Salaman Abedi, detonated an explosive device in an area of the Manchester Arena, United Kingdom. The blast killed over 20 people and injured over 100 others. Evidence shows that, from a very young age, Abedi was a “party animal” who heavily consumed marijuana. Furthermore, he was described as a person who would start fights in the street for no reason, would act rude, and would refuse to do his homework in school. He was also described as a “very slow, uneducated and passive person” who displayed aggressive tendencies. Eventually, he began shutting himself off from other people, started becoming more violent, and started showing paranoia by making statements against western societies and hanging out with dangerous crowds. Evidence suggests that this paranoia, furthered with aggressive tendencies, led to Abedi’s suicide bombing attack that day [1,13].

On May 18, 2017, Richard Rojas purposely drove a car along three blocks of pavement in New York’s Times Square, killing a teenager and injuring 22 other people. Evidence indicates that Rojas was a heavy marijuana user. He admitted on the consumption of spiced-up marijuana right before committing the attack. Further, the record show that Rojas suffered from paranoia and hallucination, which have led him to make odd statements and partake in actions that negatively affected him in the work place or while interacting with others. Paranoia and hallucination caused him to “hear voices” that led him to commit that attack [2,14].

On November 23, 2016, Arcan Cetin carried out a mass shooting that killed five people and injured many others at the Cascade Mall in Washington. Evidence indicates that Cetin was a heavy marijuana consumer. Further, he had a past of violent behavior, with some incidents including the consumption of marijuana. Although doctors prescribed him medicine for Attention Deficit Hyperactivity Disorder and other mental health issues such as Post Traumatic Stress Disorder, anxiety, and depression, he stopped taking the medicine and substituted it with by excessively consuming marijuana. This led to aggravate his mood swings and being more violent. Before the shooting, he had threatened an ex-girlfriend who lived out of state. Evidence indicates that at, while committing the attack, Cetin was shouting a woman’s name [15,16,17].

On July 26, 2016, Satoshi Uematsu stabbed to death at least 19 people and injured at least 26 others at a care facility in Sagamihara, Japan. Months prior the shooting, Uematsu suddenly started talking and acting strangely to his coworkers, who feared he could harm someone. Consequently, Uematsu tested positive for marijuana and was diagnosed with marijuana-induced psychosis and paranoid disorder after he delivered an ominous euthanasia letter to a Japanese House of Representative and telling his co-workers and the police that he intended to kill disabled people. Although he planned the attack in detail, evidence suggests that he later seemed to showed remorse and stated that “There was something wrong with [him]”. These kinds of behaviors suggest that he was suffering from psychosis and paranoia since he was in the delusion that his acts would contribute to the Japanese society and the world [18,19].

On November 27, 2015, Robert Dear killed three people and injured nine others when he carried out a mass shooting in a Planned Parenthood clinic in Colorado Springs. Dear, along with many other users, moved to Colorado after the state legalized the recreational use of marijuana. Dear was a heavy user who was described by family and friends as “an angry and occasionally violent”, and “deeply disturbed”, individual who suffered from paranoia and mood swings. Moreover, he was described as a lonely religious extremist who had a history of domestic violence against his ex-wives, who gambled, and who committed adultery on multiple occasions. About a year before the shooting, he moved to Colorado where he lived in dire conditions at a squalid trailer without running water or electricity [20,21,22].

On July 16, 2015, Muhammad Youssef Abdulazeez killed five people and injured a couple of others in his drive-by shooting at a military recruiting center in Chattanooga, Tennessee. Prone to depression and manic episodes, he started smoking marijuana heavily in high school. This addiction was going on for many years and led his mental state to deteriorate further and cause him to fail a drug test at work. Further, he started writing suicidal notes to himself and was pulled over by a police officer for driving under the influence of marijuana and alcohol. Up until the shooting, evidence indicates that Abdulazeez had a hard time keeping a job because of his manic depressive/bipolar disorder and drug use [23,24].

On June 17, 2015, 21-year-old Dylan Roof murdered nine people who were attending a prayer service in a Church in Charleston, South Carolina. He claimed that his intentions were to start a race war. His acts were preceded by years of drug abuse. Reports reveal that Roof’s drug abuse started when he was 12 years old when he would smoke marijuana three times a day. When he was 16 years old, he tried to stop smoking marijuana after telling people that his daily marijuana usage caused him to be paranoid and hear voices. According to experts, Roof started suffering panic attacks when he was 16. Nonetheless, multiple accounts claim that he kept smoking marijuana and started abusing other drugs and alcohol. During his arrest for the Charleston shooting, Roof told police officers that he abused drugs before committing such heinous act [5,25,26].

On August 9, 2014, Michael Brown was fatally shot after a physical altercation with a police officer in Ferguson, Missouri. The autopsy and toxicology report revealed that Michael Brown had THC concentration of marijuana in his blood and urine. He had five nanograms of THC on his system, which causes approximately the same level of impairment as a 0.08 percent of blood alcohol level. That much THC notably impairs someone’s judgment and perception of the surrounding environment, which may lead to anxiety and paranoia. Evidence indicates that Brown tried to reach for the officer’s gun during the altercations, which led to the officer shooting at him in close range. Thus, evidence suggests that Brown’s behavior was most likely caused by paranoia [27,28].

On April 15, 2013, Dzhokhar Tsarnaev and his brother Tamerlan, killed three people and injured over 250 by detonating homemade pressure cooker bombs near the finish line at the Boston marathon. Both brothers were heavy marijuana users since they were young teenagers. Tamerlan was killed in a police shootout following the bombings and Dzhokhar was eventually apprehended by law enforcement officers. Several acquaintances and friends knew about both brother’s marijuana consumption and sales. One of Dzhokhar friend testified that he sold marijuana to Tsarnaev days before the Boston Marathon Bombings. Unrelated to the bombings, one of Tamerlan’s friends implicated Tsarnaev in the killing of three men whose bodies were found sprinkled with marijuana. Multiple accounts noticed an increase of violent behavior from Dzhokhar for some time leading up to the bombings [29,30,31,32].

On January 8, 2011, Jared Loughner shot and killed six people, while also injuring 14 others at the then-US Representative Gabrielle Giffords’s constituent meeting held in Tucson, Arizona. Although friends and acquaintances described him as an “awkward but friendly” young man, they started noticing his behavior drastically change in college. In high school, Loughner smoked marijuana on most days. Moreover, he also started immersing himself in conspiracy theories displayed paranoia. He dropped out of high school during his final year, but was able to attend a community college. Some college peers described him becoming mentally unstable by saying and doing things that were frightening. Other peers feared that he would do something like what he actually did. He was suspended from college and never returned. Subsequently, he tried to join the army but he was rejected because he failed a drug test. Consequently, he engaged in paranoid behavior that led to him to engage in anti-government speech and target then-Representative Giffords during her constituent meeting [33,34].

These are among the many nationally reported violent cases that have, among others factors, a common root to what led these young people to commit acts of violence at the detriment to society as a whole: the extensive use or abuse of marijuana. In recent years, many States within the United States, as well as some other countries around the world, have decriminalized or legalized the recreational use of marijuana [8,12,13,19].

### 2.2. Paranoia: Marijuana Induced

In the cases above mentioned, one of the recurring conditions that most likely led perpetrators to commit violence was paranoia. Paranoia is defined by the medical dictionary as “an unfounded or exaggerated distrust of others, sometimes reaching delusional proportions”. Paranoid perceptions can co-occur with various other mental conditions as well, such as depression and dementia, and can be divided in three different psychological disorders: paranoid schizophrenia, delusional disorder (persecutory type), and paranoid personality disorder (PPD). All three conditions are similar in the sense that they all contain an “unreasonable fear” or “unreasonable belief” as the root of each condition. Hallucinations are also a common symptom on individuals who suffer from paranoia. Nonetheless, paranoia is also a likely side effect deriving from the consumption of marijuana, as well as other drugs and alcohol [3,18,31,35].

In the cases above mentioned, one of the recurring conditions that most likely led perpetrators to commit violence was paranoia. Paranoia is defined by the medical dictionary as “an unfounded or exaggerated distrust of others, sometimes reaching delusional proportions”. Evidence suggests that paranoia was among the factors that contributed in the actions of Anthony Comello, Salem Abedi, Richard Rojas, Satoshi Uematsu, Robert Dear, Dylan Roof, Michael Brown, and Jared Lougher. Each one had, in respective degrees, unreasonable beliefs. Comello admittedly shot Cali because he feared Cali had a gun and was about to shoot him. Abedi displayed paranoid beliefs while making statements against Western societies. Rojas’ paranoia was displayed in his statements and action witnessed by his coworkers; also, he claims he heard voices that led him to commit the attack. Uematsu was diagnosed with Paranoid disorder and psychosis, which led him to have delusion beliefs that his despicable acts would make contributions to society. Dear was described as a lonely religious extremist but also had a history of domestic violence, gambling, and adultery. Roof wanted to start a race war. Brown was likely paranoid about his surroundings based on the report. Lougher was suffering from paranoia and was immersing himself with conspiracy theories. Many of these tragedies are committed by individuals who were paranoid and were consuming marijuana. It is very likely that marijuana played an active role in these people’s paranoia, considering that the chemical composition of the drug has compounds that alter a person’s perception of reality as mentioned below (Table 1, [17,36,37,38]).

### 2.3. Psychosis: Marijuana Induced

Another condition that is commonly present in cases like the above are psychotic conditions. Psychotic conditions affect an individual’s mind in a way that causes that individual to experience loss of contact with reality. During a psychotic episode, the perception of reality is altered to the point where an individual is unable to distinguish reality from hallucinations. Psychotic individual can also experience delusions (false beliefs), incoherent speech, inappropriate behavior, depression, anxiety, sleep problems, social withdrawal, lack of motivation, and difficulty functioning overall [16,17,37].

In the above-mentioned cases, Uematsu was diagnosed with marijuana-induced psychosis. His coworkers’ testimony that he would talk and act inappropriately, his paranoia, and his delusion that killing patient at a care facility would benefit the society as a whole, demonstrates that he was suffering from psychotic conditions that made him lose contact with reality and led him to commit such acts. Similar symptoms were also present in cases where perpetrators acted with delusional beliefs, such as: Abedi, who suddenly started making inappropriate statements against Western societies; Dear, who was a lonely religious extremist but also had a history of domestic violence, gambling, and adultery, which strongly indicates that he was delusional, incoherent, and lost contact with reality; Rojas, Cruz, and Roof who suffered from hallucinations while having consumed large amount of marijuana throughout their lives.

Often, individuals who suffer from pre-existing medical conditions use marijuana in an attempt to alleviate their conditions. The man in Rustavi, Cruz, Kelley, Cetin, Abdulazeez, and Cruz also consumed marijuana because they were under the illusion that it would help them cope with their conditions, whether those conditions were likely induced by marijuana or not. However, it ended up worsening their conditions as time went by. What individuals are unaware of when it comes to self-medicating, is that the marijuana they consume does not have compounds that alleviate their pain or conditions; the marijuana they consume has many compounds that negatively alter their perceptions, which leads to worse conditions (Table 2, Table 3, [2,9,10,12,39,40,41]).

## 3. Discussion

### 3.1. Marijuana: General Facts

Marijuana is the most consumed illicit drug in the world, with cannabis use and dependence continuing to increase over the past two decades as the trend of legalization persists. According to the United Nations Office on Drug and Crime, over 192 million of users (ages 15–64) worldwide regularly consume marijuana, with a lifetime use of 20% of the World’s population and a significant number of individuals regularly consuming the drug [42]. In a 2017 study by the Global Burden of Disease, it estimated the age-standardized rate of cannabis use disorder, or CUD, was 289.7 per 100,000 population (95% Uncertainty Interval (UI) 248.9–339.1), affecting 22.1 million people (95% UI 19.0–25.9 million) [42]. The United States and Canada were in fact found to have among the highest age-standardized rates of CUDs in the world [42].

Cannabis is a complex plant that is made up of 400 chemical entities of which more than 60 are cannabinoid compounds, with delta-t-tetrahydrocannabinol and cannabidiol being the major compounds. Some of those cannabinoid compounds tend to have opposing effects as they affect a very important neurotransmitter system called endocannabinoid system [2]. Moreover, some cannabinoids bind to central cannabinoid receptors to control many behavioral functions, such as aggression. Furthermore, the delta-9-tetrahydrocannabinol (THC) is the chemical responsible for the intoxicating effects on individuals who consume marijuana. The THC level determines the potency of marijuana and high levels of THC likely lead to higher negative health consequences [43,44,45].

Researchers refer to marijuana having a “high potency” when it has a THC level of more than 10%. In the past years, the THC of confiscated marijuana samples rose from 3% in 1980 to 12% in 2012. Moreover, adolescents between 15 and 17 years old have reported significantly higher ED visits from 2005 to 2010, which is likely caused by the increase of marijuana potency during that time period [7]. Although THC levels that exceed 10% most likely cause serious negative health consequences, it is not uncommon to find marijuana, which THC content exceeds 20% and occasionally 230%, to be sold in places where marijuana is legalized, such as the state of Colorado [7,8]. Furthermore, while daily users refer to high potency marijuana as “the good stuff”, it is reported that daily users are five times more likely to find themselves in the hospital for psychosis symptoms such as delusions and hallucinations caused after consuming marijuana. As this paper will mention in the following pages, however, delusions and hallucinations are not the only negative effects stemming from the consumption of marijuana. Cardiovascular diseases, depression, anxiety, and violence are also among the common negative effects of marijuana [13,14,27,34,46].

### 3.2. Mental and Behavioral Changes

We apply the results of the research regarding the role of marijuana in violence. We use concepts such as personality changes, perpetrator violence, and psychosis to establish our association of marijuana with the unfortunate cases. The purpose is to illustrate negative but preventable tragic outcomes due to marijuana and its role in violence. The overall objective is to identify the role of marijuana and to suggest it is avoidable and causal nature in inducing violence [47,48,49].

In all the cases selected, marijuana use was present. For some of the individuals, marijuana use was confirmed by a physical test. In other cases, marijuana was present on their person, identifying drug use. Moreover, some individuals of the case were identified as marijuana users by outside sources.

Present in all the cases, as a result of marijuana use, was the change in personality, aggressive behavior, paranoia, and/or psychosis. All these symptoms have been documented by scientific research to be the result of marijuana use and intoxication. Another symptom, victimization, has a positive correlation with cannabis use, and the cases illustrate marijuana users and victimization. In other words, marijuana users become victims of aggression in response to their perpetration under the influence of marijuana (Table 4, [50,51]).

The DSM V provides diagnostic categories for paranoid personality, paranoia and psychosis associated with marijuana use [52].

### 3.3. Marijuana and Violence

As mentioned above, some compounds found in marijuana have an effect on central endocannabinoid receptors that control many behavioral functions, including aggression. Although there are some instances where marijuana consumption causes mild euphoria and relaxation on users, adverse acute psychopharmacological effects are very likely to occur. A study that collected data from half a century points out that even a single dose of cannabis may cause “impairments in behavioral control that may underlie impulsive, violent behavior” by altering “the normal functioning of its underlying natural substrate, the ventrolateral prefrontal cortex in man”. Furthermore, the results collected in that study provide a strong indication that chronic marijuana use suggests a possible causal effect with predicting future violence. More studies have reported that panic attacks, confusion, hallucinations, suspiciousness, and paranoia often occur in chronic marijuana users, affecting their cognition in ways that enhance aggressive responses to perceived provocations. Further, recent studies have proven causal connections between marijuana and psychosis [38,39,53].

#### Studies Show Violence and Aggression Associated with Marijuana Use

Marijuana intoxication results in panic reactions and paranoid feelings whose symptoms lead to violence [49]. The sense of fear, loss of control, and panic is associated with violence [4,54,55]. Also marijuana use increases heart rate, which may be associated with violent behavior [34,47,56,57].

When people stop using marijuana they may experience a variety of withdrawal symptoms, including sleep disturbance, irritability or restlessness, loss of appetite, anxiety, and sweating [46,58]. Experiencing any of these symptoms can make a person angry, ranging from mild irritation to violent rage. Marijuana withdrawal can lead to intimidating violent or bullying behavior, endangering the perpetrator or other people and property [59].

In incarcerated subjects, studies found that one-third of the subjects that committed homicide had used marijuana twenty-four hours before the homicide. Further, three-quarters of those subjects were experiencing at least one mental or physical effect from marijuana intoxication when the homicide occurred.

Similarly, individuals in remote Aboriginal Australian Communities who reported current cannabis use were nearly four times more likely than nonusers to present at least once for violent trauma. Homicide offenses have been repeatedly documented to be connected to drug use, and marijuana is often one of those drugs [60].

Marijuana use is also indicative of intimate partner violence [61]. Consistent use of marijuana during adolescence was the most predictive indicator of intimate partner violence [31]. Also, marijuana use during adolescence was associated with perpetration or both perpetration and victimization by an intimate partner in early adulthood [62].

There is also a positive association between peer victimization and cannabis use in adolescents. Cannabis use is likely to be associated with perpetrator victims, those who initiate violence while using marijuana and experience retaliation to their aggressive acts. This trend suggests that cannabis use might be strongly related to outward aggression by the user [1].

Cannabis use also increases an adolescent’s own likelihood of being victimized by peers. In particular, mental effects of cannabis has the potential to decrease the ability to accurately identify, evaluate, or avoid potentially dangerous persons or situations [59].

### 3.4. Psychosis

Psychosis is defined by the medial journal as “a symptom or feature of mental illness typical characterized by radical changes in personality, impaired functioning, and a distorted or nonexistent sense of objective reality”. Psychosis causes individuals to have an impaired perception of reality, consisting of hallucinations and paranoia [2,8,16]. Consumption of marijuana also proportionally increased the risk of other mental illnesses, such as schizophrenia and other types of psychoses. These marijuana use disorders are often associated with its dependence, since a user’s brain requires more and more substance use to keep the desired euphoric effect in the brain. Thus, a user is most likely to experience withdrawal symptoms when not taking the drug. Irritability, anger, and aggression are common withdrawal symptoms that former marijuana users, or marijuana users who try to quit the consumption, experience [46]. Although marijuana advocates generally state that the consumption of the drug helps individuals who suffer from PTSD or other psychiatric conditions, studies suggest that the consumption of marijuana in patients with PTSD, and in patients following a psychiatric discharge, increases the likelihood of those patients being prone to violence compared to patients who do not consume the drug [4,37]. A 50 year-span study on adult patients in the United Kingdom indicated that continued cannabis use by an individual leads is associated with a 7-fold greater odds for commission of subsequent violent crimes. The authors of that study suggest that marijuana consumption would cause impairments in neurological circuits controlling behavior that makes a user prone to violent behavior [36].

Marijuana advocates downplay the risks associated with its unrestricted consumption by saying that the drug is safe, which is a similar approach adopted by Big Tobacco years ago to downplay the risks of smoking. Yet, despite tobacco being legal, people today are well aware of the risks associated with its consumption. Stating that consuming marijuana is safe goes against many studies and researches performed that prove negative health consequences associated with the consumption of marijuana due to the multitude of compounds present and high THC levels being consumed by individuals [9,10,11,19,21].

#### Studies Show Psychosis and Paranoia

Cannabis intoxication leads to acute psychosis in many individuals and can produce short-term exacerbations of preexisting psychotic diseases [63,64,65,66]. Cannabis use also causes symptoms of depersonalization, fear of dying, irrational panic and paranoid ideas which coincide with acute intoxication and remit quickly [67].

It was reported that 15% of cannabis users identified psychotic-like symptoms, the most common being hearing voices or having unwarranted feelings of intimidation and persecution or paranoid thoughts [38].

The potency of the marijuana has varying effects on users. A study analyzed the proportion of patients in South London with first episode psychosis attributable to high-potency cannabis use and found that the use of high-potency cannabis (skunk) confers an increased risk of psychosis compared with traditional low-potency cannabis (hash) [68].

The risk of individuals having a psychotic disorder showed a roughly three times increase in users of skunk-like cannabis (high-potency) compared with those who never used cannabis. Use of skunk-like cannabis everyday conferred the highest risk of psychotic disorders compared with no use of cannabis [69]. Potency in these studies is similar to marijuana currently available in the U.S. Direct administration of cannabis resulted in predictable increased occurrence of paranoia in comparison to those who received placebo.

Epidemiological studies showed that cannabis is the most frequently used drug among those diagnosed with bipolar disorder [70]. Studies have also shown that as the frequency of cannabis use increases, so does the risk for psychotic disorders, such as schizophrenia [71]. The investigators of Schizophrenia Commission concluded that cannabis use is the most preventable risk factor for psychosis [72,73,74,75,76,77]. High proportions of persons with schizophrenia report regular cannabis use and meet criteria for cannabis use disorder [78].

Findings suggest that activity in the basal lateral medulla is involved in marijuana-induced paranoia (state of becoming afraid of things that would normally trigger fear) [77]. That means marijuana is actually enhancing type of learning about fear, leading the brain to jump to conclusions about the mild experiences, perceiving them as scarier and more strongly connected to other scary situations than they are. This marijuana induced fear-based learning helps explain why marijuana users tend to see patterns in events that are not real, such as conspiracies [78].

In a study analyzing a college population, heavy users of marijuana displayed significantly greater impairment than light users on intentional/executive functions. This led to the conclusion that heavy marijuana use is associated with residual neuropsychological effects even after a day of supervised abstinence from the drug [53,79].

### 3.5. Public Policies

These negative effects of marijuana need to be taken into account for public policy in order to treat people with addiction and possibly avoid the tragedies above mentioned. The public should know the negative consequences associated with the compounds present within the marijuana products they consume. The current legalization push in the United States lacks prudent public policy and control over the process. A prudent public policy would be to decriminalize the drug and have its composition, creation, and distribution controlled by an agency that would keep THC levels at a minimum. Moreover, studies that try to find ways to treat individuals addicted to marijuana and ways to make the drug safer by pinpointing each compound and determine whether some compounds may indeed help people who have curable health conditions. This approach would reduce the negative effects of high THC on the human body and would decrease violence occurring during marijuana deals in the black market. Furthermore, this approach will likely decrease the violence caused by marijuana and, most importantly, it would prevent tragedies such as the ones mentioned above [8,13,31,80].

## 4. Conclusions

The main scope of this paper was to inform the general public about the relationships between marijuana and violence in the general population and in individuals with mental illnesses, as recent findings do link marijuana with cases where psychosis was present. This article is a case review and not a research study; therefore, the chief limitations regard inferences that can be made from a case study. However, the findings suggest a further need for research on marijuana and violence. The authors of this paper did not intend to take sides regarding the legalization of marijuana. The focus was public health in regards to marijuana [2,11,14,18,36].

## Figures and Tables

**Table 1:** Personality change toward aggression or violence.

Paranoid Personality Disorder
A pervasive distrust and suspiciousness of others such that their motives are interpreted as malevolent, beginning by early adulthood and present in a variety of contexts, as indicated by four (or more) of the following: Suspects, without sufficient bases, that others are exploiting, harming, or deceiving him or her. Is preoccupied with unjustified doubts about the loyalty or trustworthiness of friends or associates. Is reluctant to confide in others because of unwarranted fear that the information will be used maliciously against him or her. Reads hidden demeaning or threatening meanings into benign remarks or events. Persistently bears grudges (i.e., is unforgiving of insults, injuries, or slights). Perceives attacks on his or her character or reputation that are not apparent to others and is quick to react angrily or to counterattack. Has recurrent suspicions, without justification, regarding fidelity of spouse or sexual partner.

**Table 2:** Psychosis.

Substance-Induced Psychotic Disorder
A. Presence of one or both of the following symptoms: Delusions. Hallucinations. B. There is evidence from the history, physical examination, or laboratory findings of both (1) and (2): The symptoms in Criterion A developed during or soon after substance intoxication or withdrawal or after exposure to a medication. The involved substance is capable of producing the symptoms in Criterion A. C. The disturbance is not better explained by a psychotic disorder that is not substance-induced. Such evidence of an independent psychotic disorder could include the following: The symptoms preceded the onset of the substance use; the symptoms persist for a substantial period of time (e.g., about 1 month) after the cessation of acute withdrawal or sever intoxication; or there is other evidence of an independent non-substance-induced psychotic disorder (e.g., a history of recurrent non-substance-related episodes). D. The disturbance does not occur exclusively during the course of a delirium. E. The disturbance causes clinically significant distress or impairment in social, occupational, or other important areas of functioning.

**Table 3:** Paranoia.

Subtypes Delusional Disorder
**Grandiose type**: This subtype applies when the central theme of the delusion is the conviction of having some great (but unrecognized) talent or insight or having made some important discovery.
**Persecutory type**: This subtype applies when the central theme of the delusion involves the individual’s belief that he or she is being conspired against, cheated, spied on, followed, poisoned or drugged, maliciously maligned, harassed, or obstructed in the pursuit of long-term goals.

**Table 4:** What did the cases have in common?

Cases of Marijuana Use and Symptoms	
Case	Symptom
Anthony Comello	Paranoia
Man in Rustavi	Aggressiveness, Personality Change
Nikolas Cruz	Psychosis, Hallucinations
Devin Patrick Kelley	Aggressiveness, Personality Change
Salaman Abedi	Aggressiveness, Personality Change, Paranoia
Richard Rojas	Paranoia, Hallucinations
Arcan Cetin	Aggressiveness, Personality Change
Stoshi Uematsu	Psychosis, Paranoia
Robert Dear	Aggressiveness, Paranoia
M.Y. Abdulazeez	Aggressiveness, Paranoia
Dylan Roof	Paranoia, Hallucinations
Michael Brown	Aggressiveness, Personality Change, Paranoia
Dzhokar Tsarnaev	Aggressiveness, Personality Change
Tamerlan Tsarnaev	Aggressiveness, Personality Change
Jared Loughner	Paranoia, Psychosis
